# Preparation and in vivo evaluation of glyco-gold nanoparticles carrying synthetic mycobacterial hexaarabinofuranoside

**DOI:** 10.3762/bjnano.11.39

**Published:** 2020-03-19

**Authors:** Gennady L Burygin, Polina I Abronina, Nikita M Podvalnyy, Sergey A Staroverov, Leonid O Kononov, Lev A Dykman

**Affiliations:** 1 Laboratory of Immunochemistry, Institute of Biochemistry and Physiology of Plants and Microorganisms, Russian Academy of Sciences, Prospekt Entuziastov 13, Saratov, 410049, Russia; 2 Department of Horticulture, Breeding, and Genetics, Vavilov Saratov State Agrarian University, Teatralnaya Ploshchad 1, Saratov, 410012, Russia; 3 Laboratory of Carbohydrate Chemistry, N.D. Zelinsky Institute of Organic Chemistry, Russian Academy of Sciences, Leninsky prospekt 47, Moscow, 119991, Russia; 4 Phystech School of Biological and Medical Physics, Moscow Institute of Physics and Technology (National Research University), Institutsky per. 9, Dolgoprudnyi, Moscow Region, 141701, Russia

**Keywords:** conjugation of glycosides, gold nanoparticles, lipoarabinomannan, *Mycobacterium*, spacer aglycon

## Abstract

A number of bacterial glycans are specific markers for the detection and the serological identification of microorganisms and are also widely used as antigenic components of vaccines. The use of gold nanoparticles as carriers for glyco-epitopes is becoming an important alternative to the traditional conjugation with proteins and synthetic polymers. In this study, we aimed to prepare and evaluate in vivo glyco-gold nanoparticles (glyco-GNPs) bearing the terminal-branched hexaarabinofuranoside fragment (Ara_6_) of arabinan domains of lipoarabinomannan and arabinogalactan, which are principal polysaccharides of the cell wall of *Mycobacterium tuberculosis*, the causative agent of tuberculosis. In particular, we were interested whether the antibodies generated against Ara_6_-GNPs would recognize the natural saccharides on the cell surface of different mycobacterial strains. Two synthetic Ara_6_ glycosides with amino-functionalized spacer aglycons differing in length and hydrophilicity were directly conjugated with spherical gold nanoparticles (*d* = 15 nm) to give two sets of glyco-GNPs, which were used for the immunization of rabbits. Dot assays revealed cross-reactions between the two obtained antisera with the hexaarabinofuranoside and the 2-aminoethyl aglycon used for the preparation of glyco-GNPs. Both antisera contained high titers of antibodies specific for *Mycobacteria* as shown by enzyme-linked immunosorbent assay using *M. bovis* and *M. smegmatis* cells as antigens while there was only a weak response to *M. phlei* cells and no interaction with *E. coli* cells. The results obtained suggest that glyco-GNPs are promising agents for the generation of anti-mycobacterial antibodies.

## Introduction

A number of bacterial glycans [1–2] are specific markers for the detection and the serological identification of microorganisms [3–5] and are widely used as antigenic components of vaccines [6–23]. The use of gold nanoparticles (GNPs) [24–26] as carriers for glyco-epitopes is becoming an important alternative [15,27–48] to the traditional conjugation with proteins, synthetic polymers and other carriers [15,17,38,49–55]. Immunological properties of GNPs [25,56–57] and their use in vaccine development [58] have recently been reviewed. Glyco-gold nanoparticles (glyco-GNPs) bearing residues of tumor-associated monosaccharide Tn [37] or disaccharide Thomsen–Friedenreich (TF) [35] antigens, a tetrasaccharide of the *Streptococcus pneumonia* type-14 capsular polysaccharide [33–34,47], or lipopolysaccharides of *Burkolderia mallei* [42] have been shown to be promising vaccine candidates.

Tuberculosis (TB), which is caused by the pathogenic bacterial species *Mycobacterium tuberculosis*, remains one of the ten most common causes of death worldwide. Nearly two million people die from the disease every year [59–63]. Although *M. tuberculosis* has been extensively studied [64], TB presents an ever-growing challenge, and novel strategies for the prevention and treatment of TB are urgently needed [59]. Lipoarabinomannan (LAM) and the related arabinogalactan (AG) are two major structural components of the *M. tuberculosis* cell wall. Previous studies revealed that LAM and especially its terminal oligosaccharide fragments, conjugated with proteins [7,10,19] or monophosphoryl lipid A [23], are attractive targets for the development of carbohydrate-based anti-TB vaccines [7,10,19,23,65–66].

**Figure 1:**
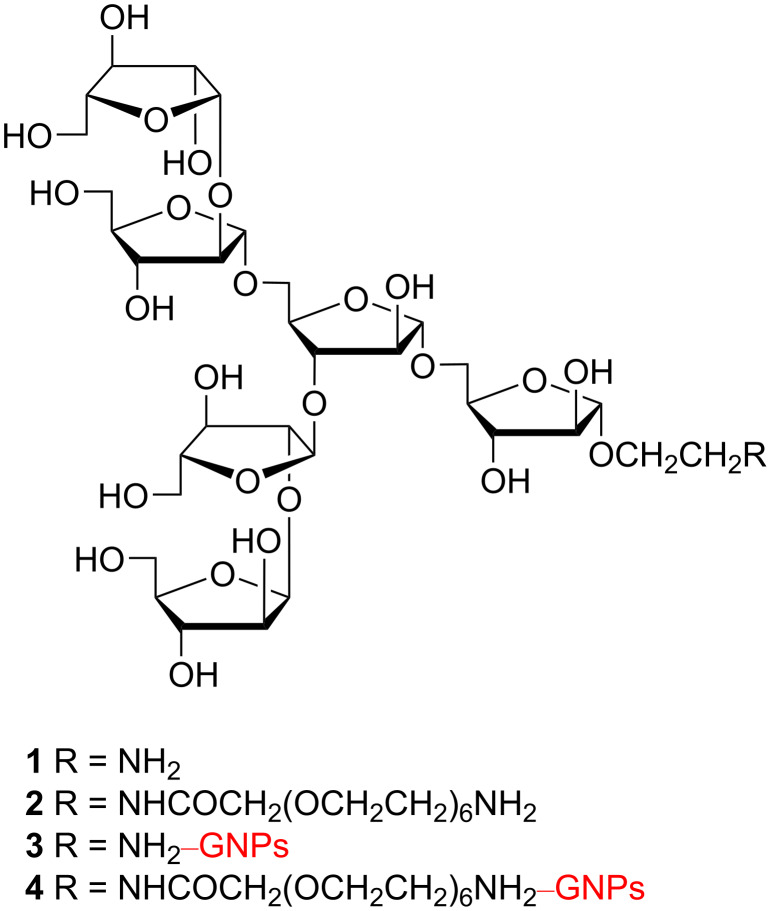
Structures of the hexasaccharide fragments of LAM/AG with amino groups at the terminal position of short (С_2_) or elongated (С_2_(ЕG)_7_) spacer aglycons (Ara_6_ glycosides **1** and **2**, respectively) and their conjugates with gold nanoparticles (GNPs) (Ara_6_-GNPs **3** and **4**, respectively).

In this study, we aimed to prepare and evaluate in vivo glyco-GNPs bearing the terminal-branched hexaarabinofuranoside fragment (Ara_6_, Figure 1), which is common for the arabinan domains of both LAM and AG and has earlier been identified as one of the lead structures [7,10,65]. Specifically, we were interested whether the antibodies generated against Ara_6_-GNPs would recognize the natural saccharides on the cell surface of different mycobacterial strains.

For the preparation of glyco-GNPs, glycans are usually transformed to glycosides with thiol-functionalized spacer aglycons that are conjugated [43–44] either with pre-formed citrate-stabilized GNPs [67] or during the in situ synthesis of glyco-GNPs [32,38,46]. It is also possible to conjugate suitably derivatized glycans [48] via additional functional groups of thiol-functionalized ligands attached to the surface of pre-formed GNPs.

We, however, chose an alternative approach that relies on the direct conjugation of amino-functionalized Ara_6_ glycosides with pre-formed citrate-stabilized GNPs that possess no additional ligands. The use of amine-terminated ligands for binding with GNPs is well established [68–71] although not very popular with glycans yet. Indeed, the direct conjugation of amino-functionalized antigenic glycans with pre-formed GNPs has not been reported yet, to the best of our knowledge. This is despite the fact that simple amines, amino acids and peptides [68–78], as well as diethylaminoethyl-dextran [79] and chitosan [80] have been reported to bind to GNPs in a pH-dependent manner as the energy of the Au–N interaction is intermediate between those of Au–S and Au–O [81]. The affinity of different functional groups to the surface of GNPs decreases in the series Au–S > Au–NH_2_ > Au–COOH [82], which enables the exchange of citrate ligands with amines. The amine-capped GNPs are stable enough to be used as targeting agents for drug-delivery applications [75], as antigens for the generation of antibodies [77], or as antimicrobial agents [78]. These nanoconjugates are nontoxic, effectively penetrate into the cells and can be used as carriers [83]. It has been demonstrated that the amine–gold surface interaction is charge-neutral and the stability of amine-capped GNPs is a finite-size effect, which is largely kinetic in origin, unlike that of thiol-capped GNPs, which are known to possess thermodynamic stability with respect to the desorption of capping ligands and subsequent particle aggregation [68].

The nature and the length of the spacer aglycon are known to affect the presentation of carbohydrate ligands [84–90]. This, in turn, determines the molecular recognition of glycan moieties including those incorporated in glyco-GNPs [38,40,91]. In order to find out whether such an influence would be critical for the immunization with Ara_6_-GNPs and specificities of the elicited antibodies, two synthetic Ara_6_ glycosides with amino-functionalized spacer aglycons differing in length and hydrophilicity (Ara_6_C_2_NH_2_ (**1**) and Ara_6_C_2_EG_7_NH_2_ (**2**) [92–93], Figure 1) were conjugated with gold nanoparticles (*d* = 15 nm) [67,94] to give two sets of Ara_6_-GNPs (**3** and **4**, respectively). The latter were used for the generation of antibodies that were then characterized by dot assay and enzyme-linked immunosorbent assay (ELISA), which confirmed their specificity against *Mycobacteria*.

## Results

### Preparation and characterization of conjugates of amino-functionalized glycosides with GNPs

#### Glycans

The known hexasaccharide fragments of LAM/AG with amino groups at the terminal position of short (С_2_) or elongated (С_2_ЕG_7_) spacer aglycons (Ara_6_ glycosides **1** and **2**, respectively, Figure 1) were prepared as described previously [92–93] and used without any further modification for direct conjugation with pre-formed GNPs.

#### Glyco-GNPs

**Figure 2:**
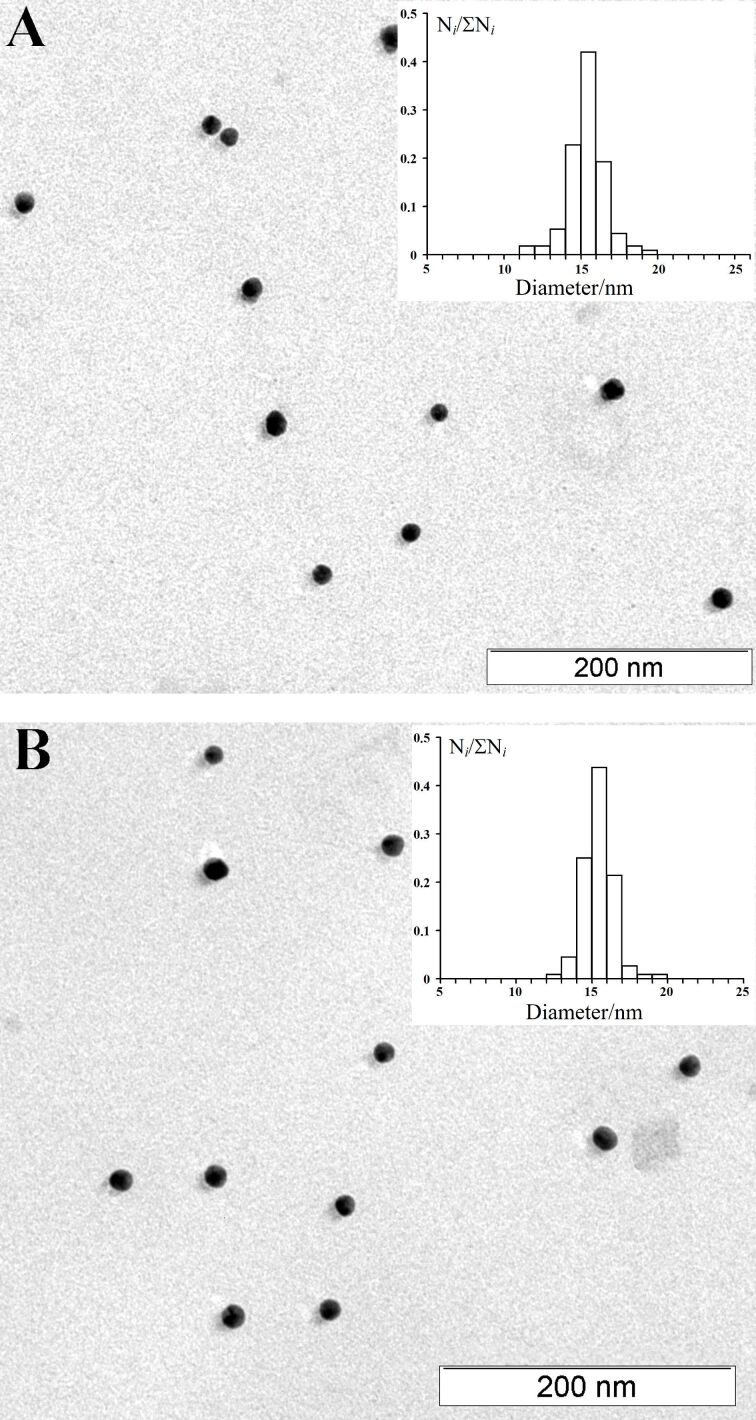
Transmission electron microscopy (TEM) images of Ara_6_-GNPs **3** (A) and **4** (B). The insets show size distribution diagrams with 15 nm average diameter in both cases.

In order to prepare the glyco-GNPs, Ara_6_ glycosides **1** and **2** (Figure 1) were directly conjugated to pre-formed [67,94] freshly prepared citrate-stabilized spherical gold nanoparticles (*d* = 15 nm, identical to those prepared earlier [77]) to give the corresponding Ara_6_-GNPs **3** and **4** (Figure 1), which have the same size as the parent GNPs according to transmission electron microscopy (TEM) (Figure 2).

According to the colorimetric determination of carbohydrates (phenol–sulfuric acid reaction, see the Experimental section for details) in the samples of glyco-GNPs **3** and **4**, which were purified from the excess of Ara_6_ glycosides **1** and **2** by centrifugation, there are no more than 100 glycoside molecules on each nanoparticle on average.

Spherical citrate-stabilized GNPs with 15 nm average diameter (which are often considered as standard GNPs for immunochemical studies due to ease of preparation and conjugation with ligands [48,56–57] and have successfully been used in our previous studies [56–57,77–78,95]) were chosen for the experiments. This is because GNPs of this size, unlike much smaller GNPs (e.g., around 2 nm in diameter), which are often derivatized in situ [32,39,46,48], can be easily prepared in advance and kept for further conjugation with a desired ligand. Another advantage of using these GNPs is their strong light absorption due to localized surface plasmon resonance (LSPR) around 520 nm while colloids of GNPs smaller than 3 nm do not exhibit an LSPR and are barely colored. It is known that the color of GNP colloids dramatically depends on size and shape of the GNPs as well as on the dielectric properties of the medium surrounding the GNPs. These features are useful in many (bio)analytical applications including monitoring their stability (as described below) [25–26,46]. Size and shape of the GNPs (spheres, nanorods, nanoshells and nanostars) were shown to influence the immunogenicity of conjugates with haptens. Large spherical GNPs (*d* = 15 and 50 nm) are the optimal antigen carriers and adjuvants for immunization [96].

Nature and length of the spacers (linkers) have been selected to modulate the stability of the Ara_6_-GNPs, as well as to control the presentation of the Ara_6_ hexasaccharide epitope on the surface of the GNPs in order to explore the effect of a linker on the immunogenicity. The very short C_2_ linker (only two aliphatic carbon atoms) of glycoside **1** was chosen to allow the Ara_6_ hexasaccharide moiety to protrude just a little above the shell of Ara_6_C_2_NH_2_-GNPs **3**. This makes the recognition of the glycan epitope potentially problematic (for this reason, such short linkers are not popular in neoglycoconjugate chemistry). Thus, glyco-GNPs **3** were initially planned to serve as negative control. The Ara_6_ hexasaccharide was also conjugated to a much longer and flexible hydrophilic linker based on heptaethylene glycol (EG_7_), which was initially expected to allow for a better recognition of the glycan epitope on the surface of Ara_6_C_2_EG_7_NH_2_-GNPs **4** than on the surface of Ara_6_C_2_NH_2_-GNPs **3**. The differences in hydrophilicity and rigidity of the spacers in glycosides **1** and **2** were also expected to affect the stability of the formed Ara_6_-GNPs **3** and **4**.

#### Stability of glyco-GNPs

Gold hydrosols are typical lyophobic colloids that are stable only under conditions of low ionic strength. Under physiological conditions, GNPs are thermodynamically unstable and require stabilization. The sol stability can be increased by coating the GNPs with a ligand layer. As a result, the particle surface acquires the properties of the stabilizing agent under use. These stabilized GNPs can be lyophilized and become much less sensitive to electrolyte-induced coagulation (due to electrostatic and hydrophobic interactions and structural-mechanical stability factors) [57,97–98]. The minimal stabilizing concentrations for both glycosides **1** and **2** were found to be 100 μg·mL^−1^. This concentration was used to conjugate antigens **1** and **2** with GNPs, which gave glyco-GNPs **3** and **4**.

**Figure 3:**
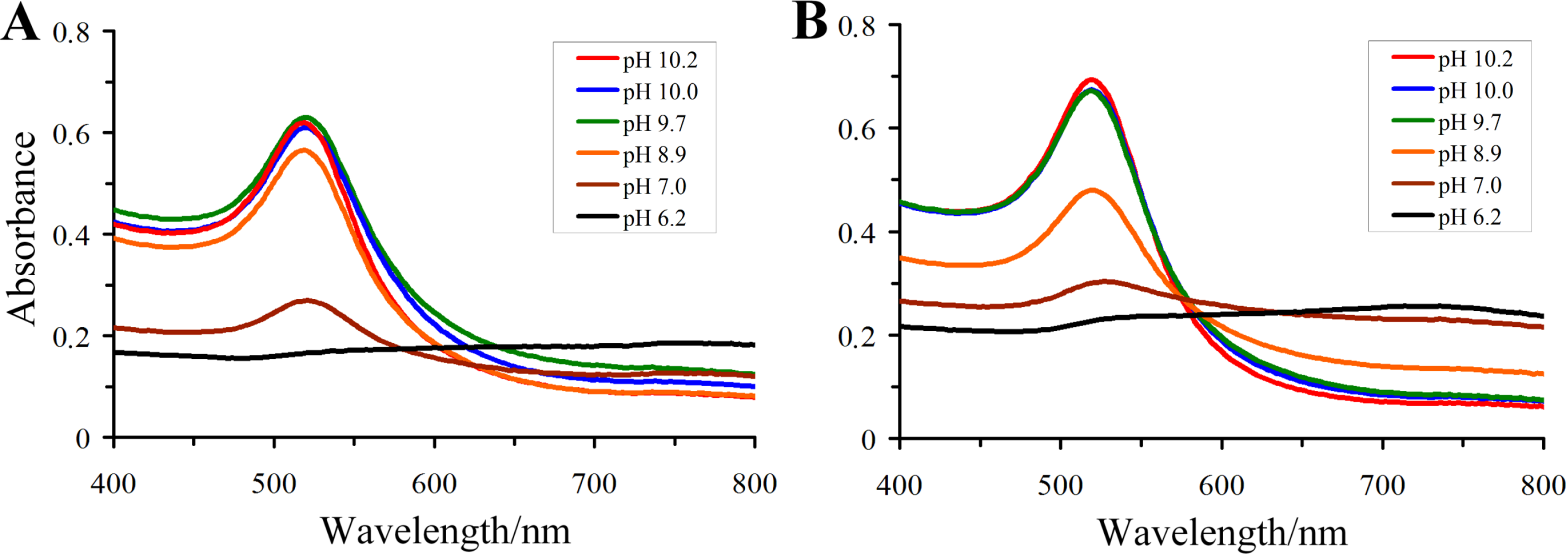
Absorption spectra of solutions of Ara_6_C_2_NH_2_-GNPs (**3**) (A) and Ara_6_C_2_EG_7_NH_2_-GNPs (**4**) (B), prepared from solutions of containing 100 μg·mL^−1^ of glycosides and 0.9% NaCl, at different рН values. The glycan components in glyco-GNPs **3** and **4** are hexasaccharide fragments (Ara_6_) of mycobacterial LAM/AG with amino groups at the terminal position of short (С_2_) or elongated (С_2_ЕG_7_) spacer aglycons (Ara_6_ glycosides **1** and **2**, respectively; see Figure 1).

The TEM data of the prepared Ara_6_-GNPs **3** and **4** (Figure 2) clearly suggest that coupling of the Ara_6_ glycosides **1** and **2** with GNPs did not change the size of nanoparticles (*d* = 15 nm) and that aggregation is absent. The addition of NaCl solution to conjugates of GNPs with Ara_6_ glycosides **1** and **2** (Ara_6_-GNPs **3** and **4**, respectively) allowed us to determine the differences in ability of Ara_6_ glycosides **1** and **2** to stabilize the GNPs at different pH values (Figure 3). The highest stabilizing ability was demonstrated by Ara_6_ glycoside **1** with 2-aminoethyl spacer aglycon while Ara_6_ glycoside **2** with a longer spacer aglycon was slightly inferior and could stabilize GNPs only at pH values of 9.7 and above. For this reason, immunization of rabbits was carried out with solutions of Ara_6_-GNPs **3** and **4** at pH ≈9.7, at which both Ara_6_-GNPs **3** and **4** were stable in saline medium.

**Figure 4:**
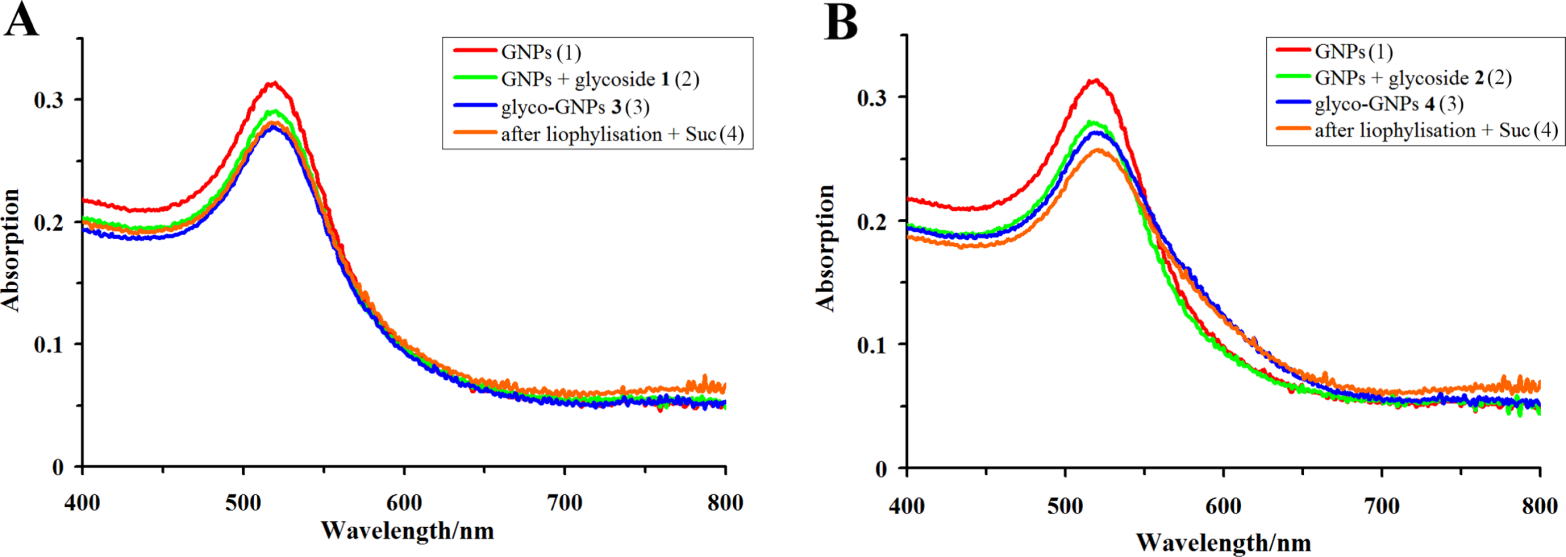
Spectra of (1) the solution of the starting GNPs prepared in water, (2) a mixture of solutions of GNPs (pH 9.7) and aqueous solutions containing 100 μg·mL^−1^ of Ara_6_ glycosides **1** (A) and **2** (B) (these mixtures were used for immunization; 0.9% NaCl was additionally present in the samples of these mixtures used for recording the spectra), (3) solutions of glyco-GNPs **3** (A) and **4** (B) prepared in water from glyco-GNPs **3** or **4**, which were purified from the excess of Ara_6_ glycosides **1** and **2** by centrifugation of the mixtures of GNPs with glycoside solutions (see (2)), and (4) solutions of glyco-GNPs **3** (A) and **4** (B) prepared in 0.9% aqueous NaCl from glyco-GNPs **3** or **4**, which were purified by centrifugation, dissolved in 5% sucrose (Suc) solution and then lyophilized.

The stability of the prepared glyco-GNPs **3** and **4** against aggregation followed from the experiments with three different types of solutions of glyco-GNPs **3** and **4**, which (1) contained an excess of Ara_6_ glycosides **1** and **2** (pH ≈9.7; these solutions were used for immunization), (2) were prepared in water from glyco-GNPs **3** or **4**, which were purified from the excess of Ara_6_ glycosides **1** and **2** by centrifugation, or (3) were prepared in 0.9% NaCl from glyco-GNPs **3** or **4**, which were purified by centrifugation, dissolved in 5% sucrose solution and then lyophilized. The absorption spectra of these solutions were virtually identical and very similar to that of the starting GNPs (Figure 4).

#### Detection of glycosides by obtained sera

**Figure 5:**
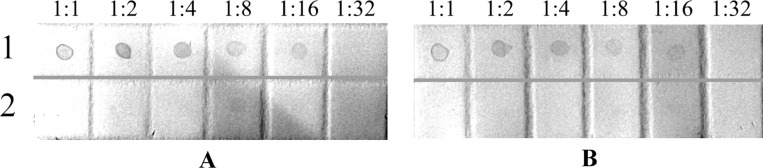
Results of the dot assay of Ara_6_ glycosides **1** (1) and **2** (2) with antisera against Ara_6_C_2_NH_2_-GNPs (**3**) (A) and Ara_6_C_2_EG_7_NH_2_-GNPs (**4**) (B). Solutions of glycosides (1 mg·mL^−1^) in H_2_O were titrated twice.

The glyco-GNPs **3** and **4** were used for the hyperimmunization of rabbits. The interaction of the obtained polyclonal rabbit antisera with the starting glycosides **1** and **2** was studied by dot assay on a polyvinylidene fluoride (PVDF) membrane (Figure 5). Ara_6_ glycoside **1** (with a short spacer aglycon) was detected by antisera against both Ara_6_C_2_NH_2_-GNPs **3** and Ara_6_C_2_EG_7_NH_2_-GNPs **4**. This means that there was a serological cross-reaction for the obtained antisera against Ara_6_-GNPs **3** and **4**. Remarkably, both antisera were equally effective in detecting Ara_6_C_2_NH_2_ (**1**) in amounts as low as 60 ng. This unequivocally proves that both antisera are specific for the Ara_6_ hexasaccharide epitope. Glycoside **2** (with a long spacer aglycon) could not be detected by the dot analysis with any of the obtained antisera.

### Interaction of the obtained sera with mycobacterial cells

**Figure 6:**
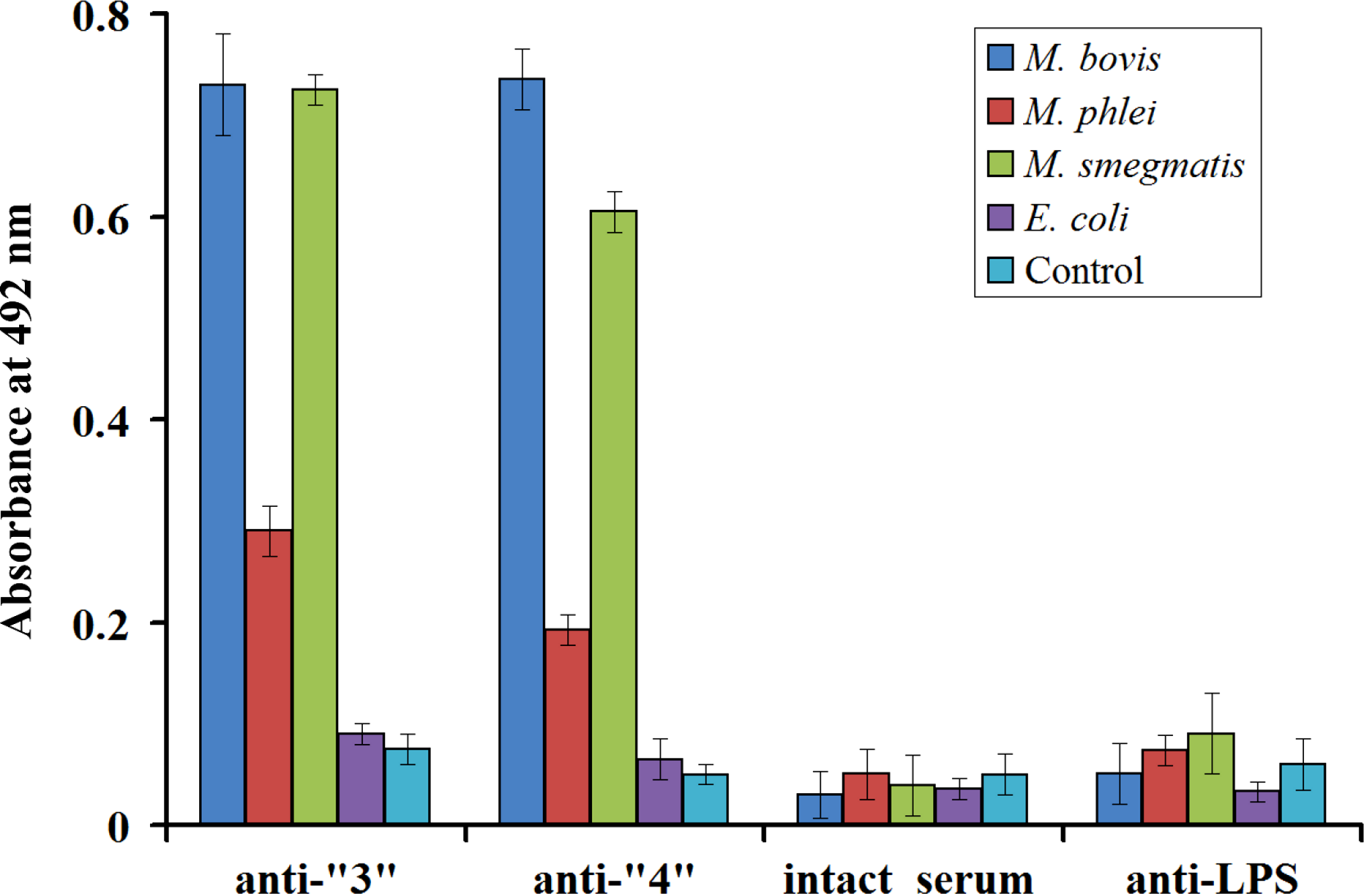
Results of ELISA of *M. bovis*, *M. phlei*, *M. smegmatis* and *E. coli* cell suspensions (10^8^ cells·mL^−1^) with antisera against Ara_6_C_2_NH_2_-GNPs **3** (anti-“**3**”), Ara_6_C_2_EG_7_NH_2_-GNPs **4** (anti-“**4**”), intact rabbit serum used for immunization or antibodies against LPS of *Azospirillum brasilense* Sp7 generated in the presence of CFA [99]. Error bars indicate the observed confidence intervals at *p* < 0.05. See also all titration curves in Figure S1 (Supporting Information File 1).

Interaction with the mycobacterial cells of three model cultures (*M. bovis*, *M. phlei* and *M. smegmatis*) has been demonstrated for both obtained antisera against Ara_6_-GNPs **3** and **4** by ELISA (Figure 6, Supporting Information File 1, Figure S1). Both antisera detected *M. phlei* cells significantly weaker than *M. bovis* and *M. smegmatis* cells. Importantly, none of the antisera interacted with *E. coli* cells. Control experiments showed that the observed specificity of the antisera against Ara_6_-GNPs **3** and **4** is due to the presence of the Ara_6_-epitope in Ara_6_-GNPs **3** and **4**. It is not related to the presence of background anti-mycobacterial antibodies in intact rabbit serum used for immunization or antibodies generated against heat-inactivated *M. tuberculosis* cells, which are present in complete Freund’s adjuvant (CFA), as no interaction of intact rabbit serum or antiserum against LPS of *Azospirillum brasilense* Sp7, generated in the presence of CFA [99], with *M. bovis*, *M. phlei*, *M. smegmatis* and *E. coli* cell suspensions could be detected (Figure 6).

## Discussion

The biosynthesis of antibodies in mammals is induced by immunogenic substances. These compounds are typically characterized by a fairly developed structure and include proteins, polysaccharides, and a number of synthetic polymers. A significant part of biologically active substances, including glycosides, has a relatively small molecular mass. Low-molecular-mass antigens belong to the category of so-called weak antigens (haptens), i.e., a pronounced immune response against them is not developed (in general, substances with molecular mass ca. 1 kDa or less are not immunogenic). As a rule, haptens are conjugated with carrier proteins, such as bovine serum albumin, hemocyanin, ovalbumin, or thyroglobulin, to allow helper T-cell epitopes to stimulate an effective antibody response [100–101]. However, in this case antibodies are formed both against the hapten and against the immunodeterminant regions of the carrier. Moreover, the use of such a carrier does not ensure the development of a pronounced immune response against weak antigens. In addition, subsequent purification and screening of the resulting antibodies are laborious and expensive, and their titer and affinity are often low. When gold nanoparticles are used as both a carrier and adjuvant, high-titer antisera are obtained that do not require further purification from ballast antibodies [56,101].

In this study, hexasaccharide fragments of LAM/AG with amino groups at the terminal position of short (С_2_) or elongated (С_2_ЕG_7_) spacer aglycons (Ara_6_ glycosides **1** and **2**, respectively; Figure 1) were conjugated with pre-formed [67,94] gold nanoparticles (*d* = 15 nm) to give the corresponding glyco-GNPs with pendant Ara_6_ moieties (Ara_6_C_2_NH_2_-GNPs (**3**) and Ara_6_C_2_EG_7_NH_2_-GNPs (**4**), respectively), which were found to be stable in the presence of 0.9% NaCl only in alkaline media (pH 8–10, Figure 3). Noteworthy is the better stabilization of GNPs by Ara_6_ glycoside **1** with a short 2-aminoethyl spacer aglycon.

It is important to stress that the removal of excess Ara_6_ glycosides **1** and **2** from solutions of glyco-GNPs (**3** and **4**) or lyophilization of the purified glyco-GNPs **3** and **4** (provided that they were dissolved in 5% sucrose solution prior to lyophilization) does not impair the stability of the prepared glyco-GNPs **3** and **4** against aggregation as follows from the similarity of the absorption spectra (Figure 4).

The prepared glyco-GNPs with pendant Ara_6_ moieties (Ara_6_-GNPs **3** and **4**) were used for the immunization of rabbits. The alkalinity of the immunogen (conjugate of an antigen with GNPs) is typical for the immunization of animals, starting with the first published work on the production of antibodies using colloidal gold [102]. The prepared Ara_6_-GNPs **3** and **4** were not separated from the excess of ligands **1** and **2** and were used for hyperimmunization of rabbits without additional purification since it was reported that the presence of excess soluble antigen along with the same antigen immobilized on GNPs may be vital for inducing high levels of antibody response in immunization [98]. Although the role of the admixture of soluble antigen in inducing protective immunity is unclear yet [98], these results might suggest that leaching of antigen from the prepared glyco-GNPs could be beneficial for the success of immunization while immunization with non-conjugated low-molecular-mass haptens **1** or **2** is not expected to induce any noticeable immune response (as noted above; see also the subsequent discussion on the putative mechanism of immunogenicity of glyco-GNPs). This also suggests that the increased stability of glyco-GNPs does not necessarily mean better immunization. Although one can argue that amine-linked carbohydrate ligands present in Ara_6_-GNPs **3** and **4** might be exchanged with various thiols present in vivo, immunization with the prepared Ara_6_-GNPs **3** and **4** was successful, which indicates that such glyco-GNPs preparations are indeed capable of inducing antibody response. Clarification of these complex issues clearly requires further studies.

The specificity of the obtained antisera against Ara_6_-GNPs **3** and **4** was then studied. Dot assay, which is a traditional way for an initial rapid assessment of specificity of the antisera obtained, revealed cross-reactions for the two obtained antisera with the parent hexaarabinofuranoside **1** with 2-aminoethyl aglycon (Figure 5). Therefore, antibodies in high titers of approximately the same specificity against the Ara_6_ hexasaccharide epitope are produced by immunization of rabbits with both Ara_6_-GNPs **3** and **4**. Of the two Ara_6_ glycosides, only glycoside **1** with a short spacer aglycon can be used to detect specific antibodies by the dot assay. Lack of interaction between glycoside **2** and the obtained antisera in the dot assay is apparently related to the desorption of glycoside **2** with the much more hydrophilic spacer aglycon from the PVDF membrane during the assay. This explanation is further supported by the fact that it was impossible to use a nitrocellulose membrane for dot assay of the glycosides because of the higher hydrophilicity of this material than that of PVDF. For both glycosides **1** and **2**, there was no adsorption to the nitrocellulose membrane, and as a consequence, no reaction with specific antisera (data not shown). The combination of these observations makes it possible to conclude that it is the Ara_6_ hexasaccharide epitope that determines specificities of both antisera.

Both antisera contained high titers of antibodies specific for *Mycobacteria* as shown by ELISA using *M. bovis* and *M. smegmatis* cells as antigens. The antisera interacted weakly with *M. phlei* cells and there was no interaction with *E. coli* cells. Control experiments showed that the observed specificity of antisera against Ara_6_-GNPs **3** and **4** is due to the presence of the Ara_6_-epitope in Ara_6_-GNPs **3** and **4** and is not related to the presence of background anti-mycobacterial antibodies, which could be present in intact rabbit serum used for immunization, or antibodies generated against heat-inactivated *M. tuberculosis* cells, which are present in CFA (Figure 6, Supporting Information File 1, Figure S1). The importance of using CFA for the generation of hapten-specific antibodies has been recently demonstrated [77]. The titers of antibodies against haptens amine-linked to GNPs decreased in the following order: (hapten + GNPs + CFA) > (hapten + GNPs) ≫ (hapten + CFA). No antibodies against GNPs have been detected in [77].

The results obtained suggest that glyco-GNPs bearing oligosaccharide fragments of mycobacterial LAM/AG are promising agents for the generation of anti-mycobacterial antibodies. The positive reaction of the obtained antibodies to the cells of all three mycobacterial cultures tested, in contrast to *E. coli* cells, confirms the antigenic identity of the Ara_6_ epitope and surface cell antigens for all *Mycobacteria* and the specificity for this group of bacteria. Nevertheless, we have identified differences in the interaction of antibodies, obtained with Ara_6_-GNPs, with the cells of three mycobacterial cultures by using the ELISA assay that may indicate a different presentation of LAM/AG on the surface of *M. bovis*/*M. smegmatis* cells and *M. phlei* cells. This observation suggests the possibility of serological individuality of *M. phlei*, despite the fact that *M. smegmatis* and *M. phlei* are phylogenetically close species. Both species belong to the group of “rapidly growing *Mycobacteria*”, in contrast to *M. bovis*, which belongs to “slowly growing *Mycobacteria*” [103]. A serological cross-reaction between the *M. bovis* and *M. smegmatis* cells (in contrast to *M. phlei* cells) was previously noted for antibodies obtained against a tuberculin-GNPs conjugate [96].

Both sets of Ara_6_-GNPs (**3** and **4**) that contained Ara_6_ glycan epitopes with different spacer aglycons were equally effective in the generation of antibodies in rabbits. The fact that even Ara_6_C_2_NH_2_-GNPs (**3**) with a very short linker (only two carbon atoms) performed well is rather unexpected since it is commonly believed that a rather long spacer aglycon (more than five carbon atoms) is required for a correct recognition of glycan moieties and efficacious immunization [38,40,46,91].

The observed differences in specificity of antibodies generated against the two sets of Ara_6_-GNPs (**3** and **4**) suggest a substantially different presentation of the same glycan on the synthesized Ara_6_-GNPs **3** and **4**. The antibodies against Ara_6_C_2_EG_7_NH_2_-GNPs **4** with the longer С_2_ЕG_7_ spacer aglycon are noticeably more selective towards different species of *Mycobacteria*. We hypothesize that this different presentation of the Ara_6_ epitope could include a different degree of clustering of glycans on the surface of the GNPs. Ara_6_C_2_EG_7_NH_2_-GNPs **4** is apparently more dispersed over the surface of the GNPs and, hence, more accessible for interaction. These unusual features of the Ara_6_-GNPs conjugates could be related to differences in structures of the solutions [104] of the parent amino-functionalized Ara_6_ glycosides **1** and **2**. We have recently argued [105–107] that the sometimes observed profound influence of the nature of the (functionalized) aglycon in a glycosyl acceptor may be related to the formation of reaction solutions with modified structures featuring different supramolecular assemblies of the reagents (supramers [104]) in solution. In a similar fashion, solutions of Ara_6_C_2_NH_2_-GNPs **3** with the shorter and more hydrophobic С_2_ spacer aglycon might form tighter [108–110] supramers in aqueous solution [104,111–112], which eventually would form more clustered Ara_6_-glycan domains on the surface of Ara_6_-GNPs. The results obtained clearly suggest that the choice of the spacer aglycon may be critical for the specificity of antibodies against the corresponding glyco-GNPs and the studies in this direction seem promising.

Glycoside **2** was previously used for conjugation with mycobacterial proteins for the serological detection of antibodies against *M. tuberculosis* [92]. It was shown that the conjugation of the recombinant proteins MPB-64 and Rv0934 with glycan **2** containing the Ara_6_ epitope increased the sensitivity of serodiagnosis by 10–15% as compared to the use of unmodified proteins. Here, instead of using protein carriers, conjugation of glycans with GNPs, which is experimentally much easier, was carried out. The obtained Ara_6_-GNPs **3** and **4** could potentially also be used for detecting antibodies against *M. tuberculosis*. We demonstrated the immunogenicity of Ara_6_-GNPs, which opens the possibility of using similar glyco-GNPs in a new generation of vaccines aimed at preventing human and animal tuberculosis. Antibodies obtained against Ara_6_-GNPs could also be used to detect *Mycobacteria* (serodiagnosis) and treat the diseases caused by them (passive immunity).

The generation of antibodies against carbohydrate antigens (epitopes) of *Mycobacteria* linked to GNPs has not been reported to the best of our knowledge. The successful use of glyco-GNPs as vaccines has been described by Parry and co-workers [37]. The authors observed that these nanomaterials generated a strong and long-lasting production of antibodies that are selective to the Tn-antigen glycan and cross-reactive toward mucin proteins displaying Tn. Other authors [33,42,47] used glyco-GNPs to prepare specific antibodies against carbohydrate antigens or epitopes of the bacterial pathogens *S. pneumonia* and *B. mallei* in combination with peptides or proteins that activated the immune response. Safari et al. [33–34] showed that the immunization of mice with glyco-GNPs containing the tetrasaccharide epitope *S. pneumonia* type 14 without T-helper peptide did not result in the activation of the immune response of the animals (mice) or the production of specific antibodies.

The situation is rather complex as our analysis of the literature data on the use of thiol- and amino-containing glyco-GNPs suggests. It appears that the efficacy of antibody generation inversely correlates with the stability of the glycan–GNPs conjugates. The induction of a specific immune response (i.e., the generation of antibodies) against haptens is T-cell-dependent and requires the uptake (phagocytosis), processing and presentation of epitopes on MHC class-II molecules by antigen-presenting cells to the specific T cell. It remains unclear how this process can proceed with a hapten. At the moment, we cannot explain the mechanism of hapten release in immune cells. We speculate that phagocytosis of the antigen and the subsequent presentation of the epitopes on the surface of macrophages are important events in this positive regulation. According to the literature data, GNPs contribute to the penetration of antigens into phagocytic cells [56]. GNPs, in addition to their adjuvant properties, could lead to a more active uptake of glycans incorporated in glyco-GNPs since free glycans (haptens) cannot be phagocytosed per se due to their small size. Probably, the substitution of glycans on the surface of glyco-GNPs with endogenous cellular thiols and amines facilitates the subsequent translocation of glycans to the surface of macrophages, which is required for the activation of specific B-cells. Clearly, such a substitution is more favored for glyco-GNPs based on amine-terminated glycans than for the more stable glyco-GNPs based on thiol-terminated glycans (for B-cell activation, the latter require the addition of peptides/proteins). A low-molecular-mass glycan (hapten) alone, i.e., without carrier, cannot induce a cellular immune response and a possible specific immune response induced by the unconjugated hapten would be minimal and hardly detected. Fallarini et al. [113] showed that the nanoconjugates are taken up by cells and that after the uptake the sugar moieties are detached from the gold surface to be presented on the surface of the cells. We believe that a similar mechanism is implemented in our case. Clearly, this theory requires further studies to support it.

Here, we demonstrated the successful use of glyco-GNPs bearing the hexasaccharide epitope of LAM/AG for the activation of a specific immune response against carbohydrate antigens in laboratory animals (rabbits). The results are helpful in the development of synthetic protein- and peptide-free glycoconjugate vaccines based on glyco-GNPs.

It should be noted that the interaction of functionalized GNPs with cells of the immune system is still far from being understood in detail and requires further studies [56]. Experiments aimed at elucidating the mechanisms of antibody production in response to the introduction of glyco-GNPs are planned in the near future.

## Conclusion

The use of GNPs conjugated with glycoside **1** containing a terminal branched Ara_6_ hexasaccharide unit of mycobacterial LAM/AG with a short 2-aminoethyl spacer aglycon (glyco-GNPs **3**) was most effective in producing specific antibodies after immunizing rabbits. The antiserum obtained by hyperimmunization of rabbits with Ara_6_C_2_NH_2_-GNPs conjugate **3** allowed the detection of LAM/AG oligosaccharides **1** and **2** as well as cells of *Mycobacteria* with high titers.

The conjugate of GNPs with glycoside **2** containing the Ara_6_ hexasaccharide with a longer oligo(ethylene glycol) spacer aglycon (glyco-GNPs **4**) showed a weak overall efficacy. In addition to the more complex synthesis of the glycoside **2**, this glycoside is poorly absorbed on nitrocellulose and PVDF membranes, which significantly complicates the immunodetection of specific antibodies. In immunochemical tests, the obtained antibodies against Ara_6_C_2_EG_7_NH_2_-GNPs **4** did not yield better results than the antibodies against Ara_6_C_2_NH_2_-GNPs **3** containing the much simpler 2-aminoethyl spacer aglycon.

The results obtained clearly suggest that the choice of a linker between glycan and GNPs may be critical for the specificity of antibodies against the corresponding glyco-GNPs and the studies in this direction seem promising. The conjugates prepared in this study (Ara_6_-GNPs) are stable in water (after removal of excess Ara_6_ glycoside by centrifugation) or in a saline medium (either at pH ≥8.9 or after lyophilization (in the presence of 5% sucrose) of the conjugate purified from excess Ara_6_ glycoside). These features suggest that Ara_6_-GNPs (and other conjugates of GNPs with related glycans) might be useful for the immunochemical detection of antibodies against surface carbohydrate antigens of *Mycobacteria*.

In conclusion, glyco-GNPs containing fragments of LAM are promising agents for the activation of immunological reactions to *Mycobacteria* in humans and animals and generation of anti-mycobacterial antibodies. In the future, similar glyco-GNPs could be used as components of anti-tuberculosis vaccines.

## Experimental

### Preparation of amino-functionalized Ara_6_ glycosides **1** and **2**

Glycoside **1** [92–93] with 2-aminoethyl aglycon (Figure 1) was synthesized as described previously [93]. Glycoside **2** [92] with extended amino-functionalized aglycon was synthesized from 2-aminoethyl glycoside **1** by *N*-acylation with *N*-trifluoroacetylated heptaethylene glycol-based amino acid HO_2_CCH_2_(OCH_2_CH_2_)_6_NHTFA (prepared from 18-crown-6 [114]) in the presence of DMT-MM [115–116] and *N*-methylmorpholine in MeOH followed by basic deprotection as described previously [92].

### Preparation of GNPs and a study of ability of Ara_6_ glycosides **1** and **2** to stabilize nanoparticles

Gold nanoparticles (GNPs, average diameter *d* = 15 nm, Figure 2) were prepared by the citrate method of Frens [67,94]. For reduction, 0.01% aqueous tetrachloroauric acid (242.5 mL, HAuCl_4_; Sigma-Aldrich) in an Erlenmeyer flask was brought to reflux with stirring on a magnetic stirrer. Then 1% aqueous sodium citrate (7.75 mL, Fluka) was added to the flask. Reflux was continued for 30 min to give a bright red sol that contained GNPs (*d* = 15 nm) identical to those prepared earlier [77] (hereinafter GNP solution).

The ability of Ara_6_ glycosides **1** and **2** to stabilize GNPs in the presence of NaCl at different pH values was determined as described below. The pH value of the GNP solutions was adjusted to 6.2, 7.0, 8.9, 9.7, 10.0, and 10.2 by addition of 0.2 М K_2_CO_3_ aqueous solution. After that, 100 μL of an aqueous solution of Ara_6_ glycoside **1** or **2** (concentration 200 μg·mL^−1^) was added to 100 μL of the obtained GNP solutions. The mixtures were incubated at room temperature for 10 min. Then 10% aqueous solution of NaCl was added to an end concentration of 0.9% (m/v) in each solution. The spectra of the obtained solutions were registered with a Tecan Spark 10M microplate reader (Tecan, Austria) at 400–800 nm (Figure 3).

To prepare glyco-GNPs, the “gold number” (minimal amount of carbohydrate antigen that protects the sol against aggregation induced by NaCl) for the glycoside solutions was first determined. To this end, 20 μL of aqueous solutions of Ara_6_ glycoside **1** or **2** (initial concentration 1 mg·mL^−1^) was titrated twice on a 96-well microtiter plate. Each well received 200 µL of GNP solution (pH 9.7) and 20 μL of 1.7 M NaCl aqueous solution. The minimal stabilizing concentration of a glycoside was established visually by the change of color of the GNP solution from red to blue in the wells of a microtiter plate. The minimal stabilizing concentrations for both glycosides were found to be 100 μg·mL^−1^. This concentration was used to conjugate antigens **1** and **2** with GNPs.

Conjugation was done by simply mixing the components: 4.5 mL of GNP solution (*d* = 15 nm) were mixed with 125 μL of 0.2 М K_2_CO_3_ aqueous solution (the pН value of the resulting solution was ca. 9.7) and 0.5 mL of aqueous solution (1 mg·mL^−1^) of Ara_6_ glycoside **1** or **2** was added while stirring to give solutions of Ara_6_-GNP **3** and **4** (containing 100 μg·mL^−1^ of glycosides **1** and **2**, respectively), which were used for immunization. The samples of these mixtures used for spectra recording additionally contained 0.9% NaCl (Figure 4(2)).

An estimation of the glycoside content in glyco-GNPs **3** and **4** by colorimetric determination of carbohydrates using the phenol–sulfuric acid reaction [117] (as described below) suggested that there are no more than 100 glycoside molecules on each nanoparticle on average. The samples of glyco-GNPs **3** and **4**, prepared as described above, were purified from the excess of Ara_6_ glycosides **1** and **2** by centrifugation (12400*g*, 30 min, Miсrospin 12 centrifuge (Biosan, Latvia)), and the precipitate obtained was then re-suspended in a volume of water equal to the volume of the starting dispersion of glyco-GNPs. A 0.5 mL sample of this aqueous solution was successively mixed with 0.5 mL of a 5% aqueous phenol solution in a 10 mL conical test tube. Next, 2.5 mL of 95% sulfuric acid was added to achieve complete mixing of the test tube contents. The solution was allowed to cool by incubation at room temperature for 15 min. Absorbance was measured in 1 cm quartz cuvettes on a Specord 250 spectrophotometer (Analytik, Jena, Germany) at 490 nm using solutions of the parent glycoside **1** for calibration. The calculation also took into consideration that 1 mL of GNPs dispersion contains ca. 1.6 × 10^12^ particles and molecular weights of glycosides **1** and **2** are 853 g·mol^−1^ and 1174 g·mol^−1^, respectively.

Stability of the glyco-GNPs **3** and **4** (prepared as described above) against aggregation was demonstrated by the following experiments. Solutions of glyco-GNPs **3** and **4** containing 100 μg·mL^−1^ Ara_6_ glycosides **1** and **2** (for the spectra, see Figure 4(2)), were centrifuged for 30 min at 12400*g* on a Miсrospin 12 centrifuge (Biosan, Latvia) and the precipitate was resuspended in water to give solutions of glyco-GNPs with spectra (Figure 4(3)) virtually identical to the those of starting solutions of glyco-GNPs **3** and **4** (Figure 4(2)) and very similar to that of the starting GNPs (Figure 4(1)). Alternatively, the precipitate of glyco-GNPs **3** and **4** purified from the excess of Ara_6_ glycosides **1** and **2** by centrifugation (see above) was dissolved in 5% sucrose solution, lyophilized and then the residue was resuspended in 0.9% NaCl to give solutions of glyco-GNPs with spectra (Figure 4(4)) virtually identical to the those of starting solutions of glyco-GNPs **3** and **4** (Figure 4(2)) and very similar to that of the starting GNPs (Figure 4(1)).

### Transmission electron microscopy

Glyco-GNPs **3** and **4** were characterized using a Libra 120 transmission electron microscope (Carl Zeiss, Germany) at 120 kV accelerating voltage at the "Simbioz" Center for the Collective Use of the Research Equipment in the Field of Physical-Chemical Biology and Nanobiotechnology at the Institute of Biochemistry and Physiology of Plants and Microorganisms of the Russian Academy of Sciences (IBPPM RAS). Approximately 20 μL of glyco-GNPs suspension was applied to a film (Parafilm, USA) and a formvar-coated copper grid (200 mesh) was placed on the drop for 20 min. Thermal attachment was obtained through holding the grid near an incandescent lamp for 2 min. The excess of the liquids was removed by touching the grid to a strip of the filter paper. The grid was washed by a drop of deionized water, dried and then analyzed by TEM. According to TEM data, the prepared Ara_6_-GNPs **3** and **4** had the same size (*d* = 15 nm) as the parent GNPs (Figure 2).

### Animal immunization and obtaining antisera

Rabbits were immunized with the Ara_6_-GNPs **3** and **4** (one animal for each type of glyco-GNPs) as follows: 0.5 mL of solutions of Ara_6_-GNP **3** and **4** were mixed with 0.5 mL of complete Freund’s adjuvant (CFA, Sigma, USA). Polyclonal antibodies were raised by subcutaneous immunization of chinchilla rabbits with the solutions of glyco-GNPs **3** and **4** at 10 points along the spinal column, by giving four injections with an interval of 14 days between them. The obtained sera were tested for interaction with the glycosides **1** and **2** by dot assay (Figure 5) and with the mycobacterial cells by ELISA (Figure 6, Supporting Information File 1, Figure S1).

Animal care and handling were in accordance with the Guide for the Care and Use of Laboratory Animals, the European Convention for the Protection of Vertebrate Animals Used for Experimental and Other Scientific Purposes, and the legislation of the Russian Federation. The use of the animals was also approved by the institution where the experiments were performed.

### Dot assay

The specificity of the obtained antisera to both Ara_6_ glycosides **1** and **2** was tested by the dot immunoassay (titrated twice in the microtiter plate) as follows. An aliquot (1 μL) of solution of each antigen (**1** or **2**) in the double dilutions (initial concentration, 1 mg·mL^−1^) was spotted onto a Westran S polyvinylidene fluoride (PVDF) membrane (Whatman), and the membrane was incubated in a dry-air thermostat at 60 °C for 15 min. After spotting and drying, the membrane was then blocked for 12 h with 2% powdered milk diluted in 10 mM PBS. This procedure was performed to prevent a nonspecific antibody adsorption. Then the membrane was incubated in the obtained rabbit antisera, diluted 1:50 with 0.01 M PBS, at room temperature overnight. The membrane was washed four times at 15 min intervals with PBS containing 0.02% Tween 20. Then, it was incubated in a solution of peroxidase-conjugated AffiniPure Goat Anti-Rabbit IgG (H+L) (Jackson ImmunoResearch Laboratories; diluted 1:2000) in PBS containing 0.02% Tween 20 and 0.02% powdered milk for 90 min. The membrane was washed four times for 15 min with PBS containing 0.02% Tween 20. After that, the membrane was treated with a substrate mixture of 0.05% 3,3′-diaminobenzidine and 0.02% hydrogen peroxide in 0.15 M PBS until intense brown dots appeared (Figure 5).

### Bacterial cultures and growth conditions

Bacterial cultures of *M. bovis* (vaccine strain BCG), *M. phlei*, and *M. smegmatis* were obtained from the collection of pathogenic and vaccine microorganisms of animals of Kovalenko Institute of Experimental Veterinary Medicine (Moscow, Russia) [118]. A culture of *E. coli* strain K-12 was obtained from the Collection of the Rhizosphere Microorganisms of the Institute of Biochemistry and Physiology of Plants and Microorganisms RAS (Saratov, Russia) [119]. The cells were grown on a solid nutrient medium for isolation of *Mycobacteria* (the Löwenstein–Jensen medium) at 37 °C for 25 days.

### Enzyme-linked immunosorbent assay

Detection of serological reactions of antisera with mycobacterial cells was carried out by ELISA in 96-well polystyrene plates using the standard procedure, as previously described [120]. Aliquots (50 μL) of each bacterial suspension in twofold dilutions (initial concentration, 10^8^ cells·mL^−1^) were immobilized in the wells through simple adsorption, kept for 30 min on a shaker at room temperature. The samples were replaced with 100 μL of 0.05% polyethylene glycol 20000 (PEG), added to each well to block the free binding sites on polystyrene. This solution was replaced with 50 μL of primary antibodies (antisera to glyco-GNPs, or intact serum, or antiserum to LPS of *Azospirillum brasilense* Sp7 [99] as negative control) diluted 1:100 in PBS containing 0.02% Tween 20 and 0.005% PEG (for prevention of nonspecific antibody sorption). After incubation for 40–60 min, the wells were washed three times with 100 μL of PBS containing 0.02% Tween 20, and 50 μL of peroxidase-conjugated AffiniPure Goat Anti-Rabbit IgG (H+L) (Jackson ImmunnoResearch Laboratories, USA; diluted 1:2000) in PBS containing 0.02% Tween 20 and 0.005% PEG was placed in each well. After 30 min incubation, the wells were washed twice with 100 μL of PBS containing 0.02% Tween 20, and peroxidase activity was estimated by adding to each well 50 μL of a substrate mixture of 0.03% *o*-phenylenediamine and 0.02% hydrogen peroxide in 0.1 M sodium citrate buffer (pH 4.5). The enzyme reaction was stopped with 100 μL of 1 N H_2_SO_4_. The absorbance at 492 nm was read on a Multiskan Ascent analyzer (Thermo). Absorbance values at 492 nm for the wells without bacterial cells were using as the control. Data were processed with Microsoft Excel 2003 software (Microsoft Corp.); 95% confidence intervals are given (Figure 6, Supporting Information File 1, Figure S1).

## Supporting Information

File 1 Results of ELISA of *M. bovis*, *M. phlei*, *M. smegmatis* and *E. coli* cell suspensions.
