# Supplementary material for: Preparation and in vivo evaluation of glyco-gold nanoparticles carrying synthetic mycobacterial hexaarabinofuranoside
Source: Beilstein J Nanotechnol. 2020 Mar 19;11:480–93. doi: 10.3762/bjnano.11.39 (PMC7113550; doi:10.3762/bjnano.11.39)
Supplement: File 1 — Results of ELISA of M. bovis, M. phlei, M. smegmatis and E. coli cell suspensions. [file Beilstein_J_Nanotechnol-11-480-s001.pdf]

BEILSTEIN JOURNAL OF NANOTECHNOLOGY

Supporting Information

for

Preparation and in vivo evaluation of glyco-gold nanoparticles carrying synthetic mycobacterial hexaarabinofuranoside

Gennady L. Burygin, Polina I. Abronina, Nikita M. Podvalnyy, Sergey A. Staroverov, Leonid O. Kononov and Lev A. Dykman

Beilstein J. Nanotechnol. **2020,** *11,* 480–493. doi:10.3762/bjnano.11.39

Results of ELISA of *M. bovis*, *M. phlei*, *M. smegmatis* and *E. coli* cell suspensions

License and Terms: This is a supporting information file under the terms of the Creative Commons Attribution License (http://creativecommons.org/licenses/by/4.0). Please note that the reuse, redistribution and reproduction in particular requires that the authors and source are credited.
The license is subject to the *Beilstein Journal of Nanotechnology* terms and conditions: (https://www.beilstein-journals.org/bjnano)

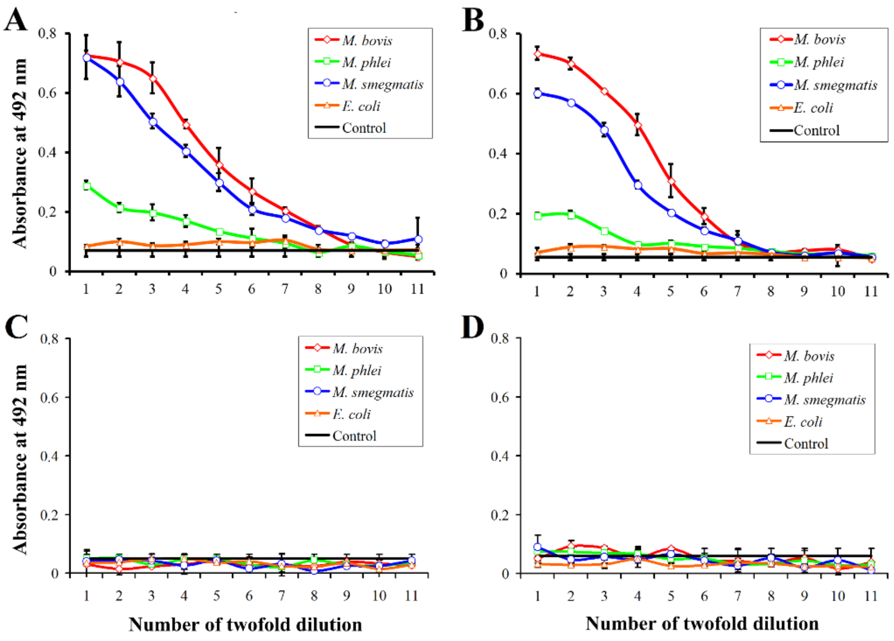

Figure S1: Results of ELISA of *M. bovis*, *M. phlei*, *M. smegmatis* and *E. coli* cell suspensions (10^8 cells·mL^{-1}) with antisera against $Ara_6C_2NH_2$-GNPs **3** (A), against $Ara_6C_2EG_7NH_2$-GNPs **4** (B), against intact serum of the rabbit used for immunization (C) or antibodies against LPS of *Azospirillum brasilense* Sp7 generated in the presence of CFA (D) [1]. Error bars indicate the observed confidence intervals at $p < 0.05$.

References

1. Konnova, O. N.; Boiko, A. S.; Burygin, G. L.; Fedonenko, Y. P.; Matora, L. Y.; Konnova, S. A.; Ignatov, V. V. *Microbiology (London, U. K.)* **2008,** *77,* 305–312. doi:10.1134/S0026261708030090
